# Compression of 3D Optical Encryption Using Singular Value Decomposition

**DOI:** 10.3390/s25154742

**Published:** 2025-08-01

**Authors:** Kyungtae Park, Min-Chul Lee, Myungjin Cho

**Affiliations:** 1 School of ICT, Robotics, and Mechanical Engineering, IITC, Hankyong National University, 327 Chungang-ro, Anseong 17579, Republic of Korea; suminyes@hknu.ac.kr; 2 Graduate School of Computer Science and Systems Engineering, Kyushu Institute of Technology, 680-4 Kawazu, Iizuka-shi 820-8502, Fukuoka, Japan; lee@csn.kyutech.ac.jp

**Keywords:** compression, double random phase encryption, singular value decomposition, volumetric computational reconstruction

## Abstract

In this paper, we propose a compressionmethod for optical encryption using singular value decomposition (SVD). Double random phase encryption (DRPE), which employs two distinct random phase masks, is adopted as the optical encryption technique. Since the encrypted data in DRPE have the same size as the input data and consists of complex values, a compression technique is required to improve data efficiency. To address this issue, we introduce SVD as a compression method. SVD decomposes any matrix into simpler components, such as a unitary matrix, a rectangular diagonal matrix, and a complex unitary matrix. By leveraging this property, the encrypted data generated by DRPE can be effectively compressed. However, this compression may lead to some loss of information in the decrypted data. To mitigate this loss, we employ volumetric computational reconstruction based on integral imaging. As a result, the proposed method enhances the visual quality, compression ratio, and security of DRPE simultaneously. To validate the effectiveness of the proposed method, we conduct both computer simulations and optical experiments. The performance is evaluated quantitatively using peak signal-to-noise ratio (PSNR), structural similarity index (SSIM), and peak sidelobe ratio (PSR) as evaluation metrics.

## 1. Introduction

Recently, information security has become one of the most critical factors across various industries, including the Internet of Things (IoT), medical devices, multimedia, defense applications, and finance. Optical encryption, a key technology in information security, has proven useful for a wide range of applications. In particular, double random phase encryption (DRPE), which utilizes a 4*f* imaging system and two distinct random phase masks, has been extensively studied [1,2,3,4,5,6,7,8,9,10]. DRPE offers strong security and high processing speed for both encryption and decryption, as it leverages optical components and light-based operations. Unlike digital encryption methods, DRPE is inherently resistant to data breaches because the random phase information is difficult to estimate or reproduce.

In the DRPE encryption process, the primary data first passes through the first random phase mask (RM1), whose phase values follow a uniform distribution in the range [0, 2π]. It then propagates through a 4*f* imaging system containing a second random phase mask (RM2), which serves as key information in the decryption process. The final output is the encrypted data. However, DRPE has a critical vulnerability: if the key information, specifically the complex conjugate of RM2, is exposed, the original data can be easily decrypted. To address this issue and several attacks on DRPE, numerous studies have been conducted [11,12].

Photon-counting DRPE [13,14,15] enhances security by applying a photon-counting imaging technique to the encrypted data. Since only a small number of photons are extracted from the encrypted data, even if the key is compromised, the original data cannot be accurately reconstructed. However, because the decrypted data cannot be visually perceived and must be detected using methods such as correlation, the 3D DRPE technique [16] has been proposed. This method incorporates integral imaging [17,18,19,20,21,22,23,24,25,26], originally introduced by G. Lippmann, along with volumetric computational reconstruction (VCR) [27,28]. In this approach, the reconstruction depth of the primary data serves as an additional key, as integral imaging and VCR enable the extraction of 3D information. Consequently, 3D DRPE improves both the security and visual quality of the decrypted data.

Nonetheless, 3D DRPE still suffers from a major drawback: the encrypted data are complex-valued and require a large amount of storage. To mitigate this issue, a 3D DRPE method for encrypting two primary data simultaneously has been proposed [29]. In this technique, two different data sets are embedded into the amplitude and phase components of the encryption process, thereby reducing the total data volume. However, since these techniques only achieve limited data reduction, a more effective compression method for DRPE is still required.

In this paper, we propose a novel compression technique for DRPE based on singular value decomposition (SVD) [30,31,32,33], which enhances both data compression and security. In the proposed method, the encrypted data are decomposed into three matrices using SVD. By retaining only a few significant diagonal entries from the diagonal matrix, the encrypted data can be effectively compressed. Furthermore, by employing integral imaging and VCR, the reconstruction depth serves as an additional key to further enhance security. Despite the compression, the visual quality of the decrypted data can still be preserved or even improved.

To demonstrate the feasibility of the proposed method, we conduct both simulations and optical experiments. For quantitative performance evaluation, we calculate the peak signal-to-noise ratio (PSNR), the structural similarity index measure (SSIM), and the peak sidelobe ratio (PSR).

This paper is organized as follows. Section 2 provides a brief overview of double random phase encryption. Section 3 introduces the application of singular value decomposition to DRPE. Section 4 describes volumetric computational reconstruction for the proposed method. Experimental results and performance analysis are presented in Section 5. Finally, conclusions are drawn in Section 6.

## 2. Double Random Phase Encryption

In this section, we present the fundamental principles of double random phase encryption (DRPE) for both encryption and decryption processes. DRPE encrypts the primary data using a 4*f* imaging system with two different random phase masks. The same 4*f* system and the complex conjugate of the second random phase mask are then used as key information to decrypt the data. Both random phase masks follow a uniform distribution in the range [0, 2π].

**Figure 1:**
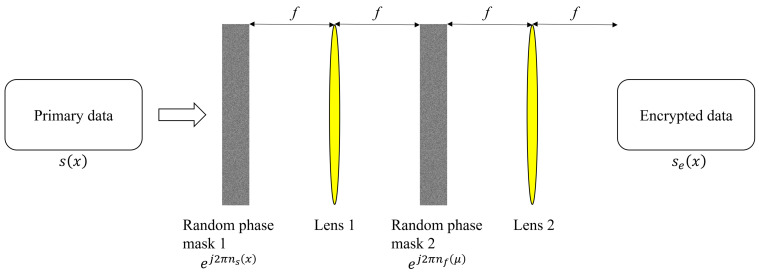
Encryption process in double random phase encryption.

Figure 1 illustrates the encryption process in DRPE. The primary data pass through the first random phase mask (RM1) in the spatial domain. Specifically, the primary data s(x) are multiplied by the first random phase mask $e^{j2\pi n_{s}\left(x\right)}$, where $n_{s}\left(x\right)$ follows a uniform distribution in the range [0, 1]. The result is then focused onto the Fourier plane of the first imaging lens (Lens 1), effectively performing a Fourier transform. At this plane, the second random phase mask (RM2) is applied as $e^{j2\pi n_{f}\left(\mu \right)}$, where $n_{f}\left(\mu \right)$ also follows a uniform distribution in [0, 1]. The signal then passes through the second imaging lens (Lens 2), which performs an inverse Fourier transform. The final output is the encrypted data, which are complex-valued. The encryption process is mathematically expressed as [1]

(1) $$s_{e}\left(x\right)=F^{-1}\left[F\left\{s\left(x\right)e^{j2\pi n_{s}\left(x\right)}\right\}\times e^{j2\pi n_{f}\left(\mu \right)}\right].$$

In Equation (1), $s_{e}\left(x\right)$ is the encrypted data, s(x) is the primary data, and $e^{j2\pi n_{s}\left(x\right)}$ and $e^{j2\pi n_{f}\left(\mu \right)}$ are the first and second random phase masks in the spatial and frequency domains, respectively. *x* and μ denote the spatial and frequency domain coordinates, while *F* and $F^{-1}$ represent the Fourier and inverse Fourier transforms.

**Figure 2:**
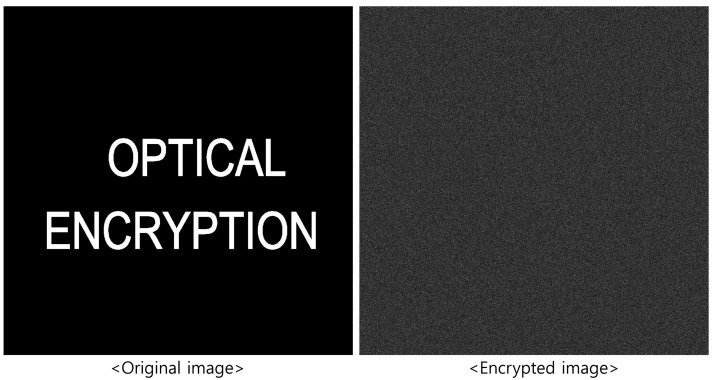
Encryption result using double random phase encryption.

Figure 2 shows the original image and the corresponding encrypted image. As shown in Figure 2, the encrypted image appears as random noise, making it impossible to visually identify the original content, thereby enhancing security. Moreover, since the process uses optical components, it operates at the speed of light.

**Figure 3:**
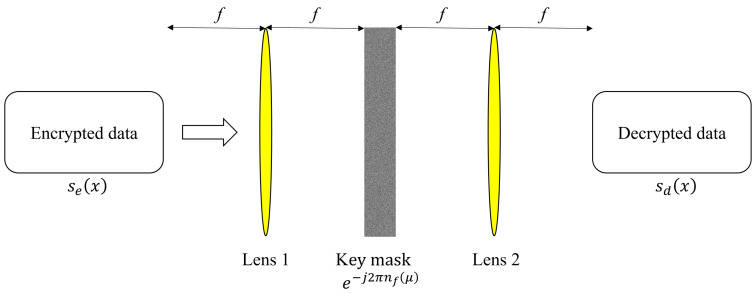
Decryption process in double random phase encryption.

The encrypted data are securely transmitted to the receiver. The decryption process is illustrated in Figure 3. First, the encrypted data are focused onto the Fourier plane of Lens 1, converting them to the frequency domain. The key information, which is the complex conjugate of RM2, $e^{-j2\pi n_{f}\left(\mu \right)}$, is then multiplied. Finally, the decrypted data are obtained through Lens 2 and captured by an imaging device such as a camera. The decryption process is expressed as [1]

(2) $$s_{d}\left(x\right)=\left|F^{-1}\left[F\left\{s_{e}\left(x\right)\right\}\times e^{-j2\pi n_{f}\left(\mu \right)}\right]\right|.$$

In Equation (2), $s_{d}\left(x\right)$ is the decrypted image. Since only the amplitude can be captured by the imaging device, the first random phase mask (RM1) is effectively canceled out.

**Figure 4:**
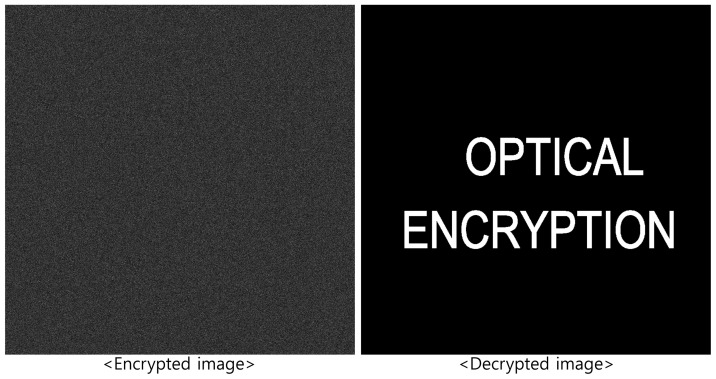
Decryption result by double random phase encryption with correct key information.

**Figure 5:**
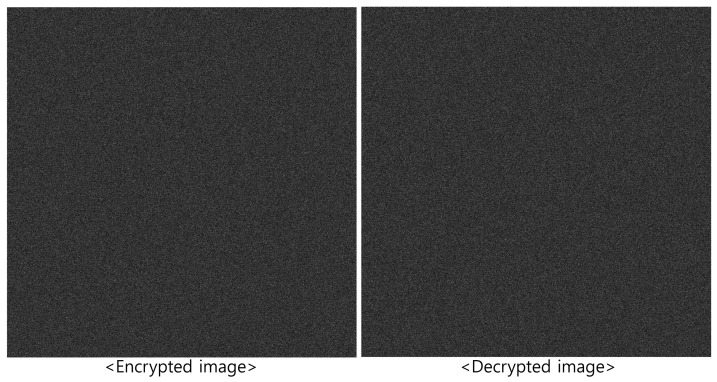
Decryption result of double random phase encryption with incorrect key information.

Figure 4 shows the decrypted image using the correct key, which matches the original image in Figure 2. Conversely, Figure 5 shows the results when incorrect key information is used, producing a noise-like image. This demonstrates the strong security performance of DRPE.

However, one drawback of DRPE is the large size of encrypted data due to their complex-valued nature. For example, if the primary data are an integer matrix of size 1500×1000 (1.5 MB), the encrypted data become a complex matrix of the same size, requiring 3 MB since each complex number stores both real and imaginary parts. As the size of the primary data increases, the encrypted data size increases proportionally. To address this issue, we propose a compression method for DRPE using singular value decomposition (SVD).

## 3. Singular Value Decomposition for DRPE

Singular value decomposition (SVD) [30,31,32,33] is a fundamental technique in linear algebra that decomposes any matrix into three simpler matrices. It is defined as [30,31,32,33]

(3) $$M=U\Sigma V^{T}.$$

Here, *U* is an m×m unitary matrix, Σ is an m×n diagonal matrix with non-negative real numbers (the singular values), and *V* is an n×n unitary matrix. The columns of *U* and *V* are the eigenvectors of $MM^{T}$ and $M^{T}M$, respectively, and are orthonormal. Since both matrices are symmetric, their eigenvectors can be chosen as orthonormal [30,31,32,33].

**Figure 6:**
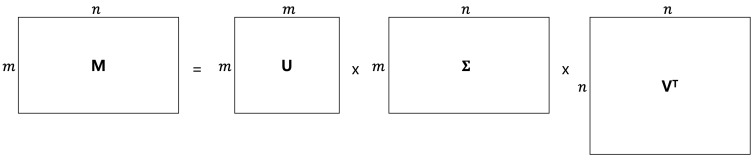
Singular value decomposition.

Figure 6 illustrates how SVD works. As shown in Figure 6, the matrix *M* can be factorized into three different matrices, namely *U*, Σ, and *V*.

In the rectangular diagonal matrix Σ, $\sigma_{i}=\Sigma_{ii}$ are singular values of matrix *M*. The number of non-zero singular values is equal to the rank of *M*, that is, r≤min{m,n}. The columns of *U* are called the left-singular vectors. The columns of *V* are called the right-singular vectors. In addition, $u_{1},\ldots ,u_{m}$ and $v_{1},\ldots ,v_{m}$ are orthogonal bases. In SVD for data compression, the number of diagonal entries in Σ, k≤min{m,n} is used. Therefore, SVD for data compression can be written as [30,31,32,33]

(4) $$A_{k}=\sum_{j=1}^{k}\sigma_{j}u_{j}v_{j}^{T},$$

where *k* is smaller than the rank of the matrix, *M*. Applying Equation (4) to the encryption process in DRPE, compressed DRPE data can be obtained.

**Figure 7:**
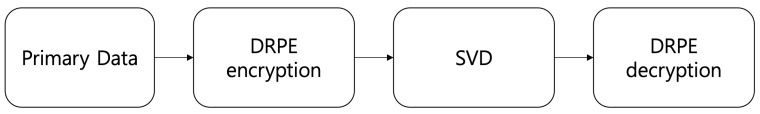
Double random phase encryption with singular value decomposition.

Figure 7 illustrates the proposed method, where the encrypted data are compressed using SVD and then decrypted using the DRPE process. The proposed method is expressed as

(5) $$\tilde{s_{d}}\left(x\right)=\left|F^{-1}\left[F\left\{SVD\left(s_{e}\left(x\right)\right)\right\}\times e^{-j2\pi n_{f}\left(\mu \right)}\right]\right|,$$

where SVD is singular value decomposition using Equation (4) and $\tilde{s_{d}}\left(x\right)$ is the decrypted data in DRPE with the SVD process.

From our proposed method, the total amount of compressed data can be calculated using the SVD process. The total amount of primary data is an integer value m×n. The total amount of encrypted data by DRPE is a complex value m×n. The total amount of compressed data by SVD is a complex value k×(m+n+1). For example, when 1500×1000 data are compressed by SVD with k=100, the total amount of primary data is 1500×1000 = 1,500,000 bytes. The total amount of encrypted data by DRPE is 1500×1000×2 = 3,000,000 bytes. The total amount of compressed data by SVD with k=100 is 100×(1500+1000+1)×2 = 500,200 bytes. It is noticed that the amount of compressed data is 3 times better than that of primary data.

**Figure 8:**
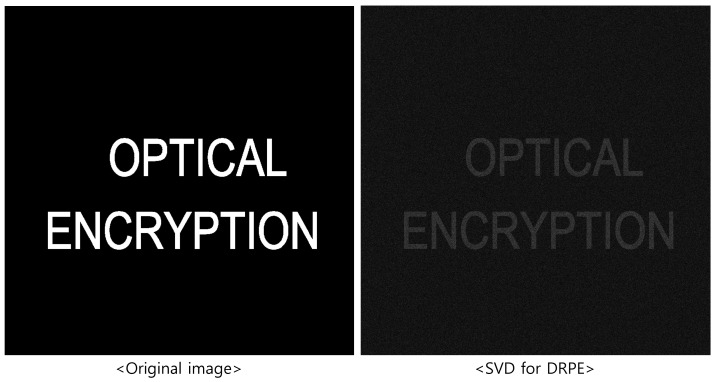
Compression result by DRPE with SVD (50 diagonal entries).

Figure 8 shows the original and decrypted images when 50 diagonal entries are used. As shown in Figure 8, the decrypted image preserves the shape of the original but appears darker due to information loss.

**Figure 9:**
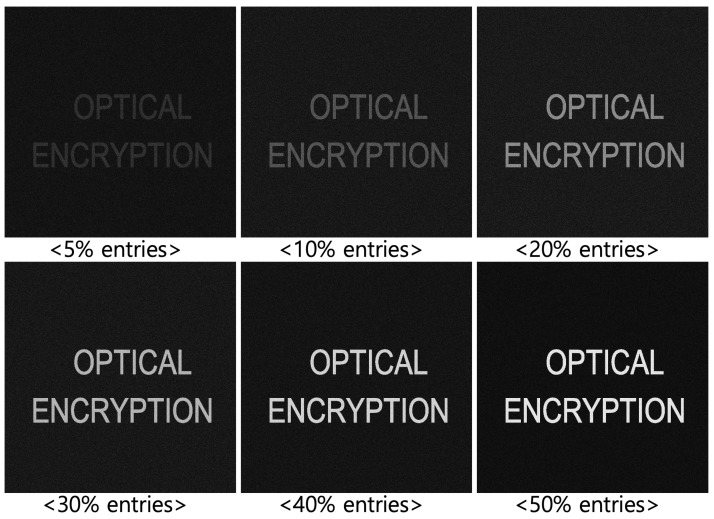
Compression results by DRPE with SVD using various diagonal entries.

Figure 9 presents results for different values of *k* (50, 100, 200, 300, 400, and 500). As *k* increases, the brightness and quality of the decrypted image improve, but the compression ratio decreases. The compression ratio (CR) is defined as

(6) $$CR=\frac{\mathrm{uncompressed}\;\mathrm{data}\;\mathrm{size}}{\mathrm{compressed}\;\mathrm{data}\;\mathrm{size}}.$$

To maximize the compression ratio, a smaller *k* should be used. However, as shown in Figure 9, a smaller *k* results in poorer visual quality. To address this trade-off, we incorporate integral imaging and volumetric computational reconstruction (VCR) in the following section.

## 4. Volumetric Computational Reconstruction for 3D DRPE with SVD

**Figure 10:**
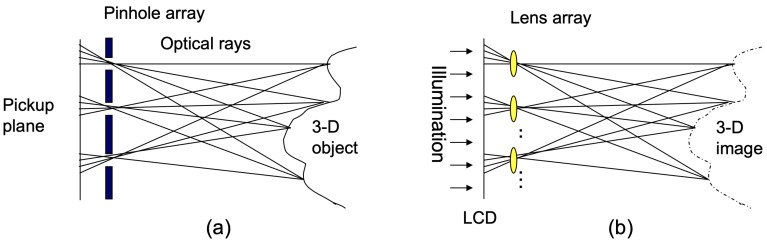
Integral imaging. (**a**) Pickup and (**b**) reconstruction.

Figure 10 illustrates the principle of integral imaging, which was first proposed by G. Lippmann [17]. Integral imaging can record multiple 2D images from different perspectives using a pinhole array, lens array, or camera array, as shown in Figure 10a. These multiple 2D images are referred to as elemental images. During the reconstruction process, the elemental images are projected into 3D space through a homogeneous pinhole or lens array, thereby generating a 3D image. This approach provides full parallax and continuous viewing points without the need for special glasses or a coherent light source.

However, when using a pinhole or lens array, the resolution of each elemental image is limited, as the total sensor resolution is divided among the pinholes or lenses. As a result, the resolution of the reconstructed 3D image is reduced. To address this limitation, it is necessary to enhance the resolution of each elemental image. In this paper, we employ synthetic aperture integral imaging (SAII) [22], which utilizes a camera array instead of a pinhole or lens array.

**Figure 11:**
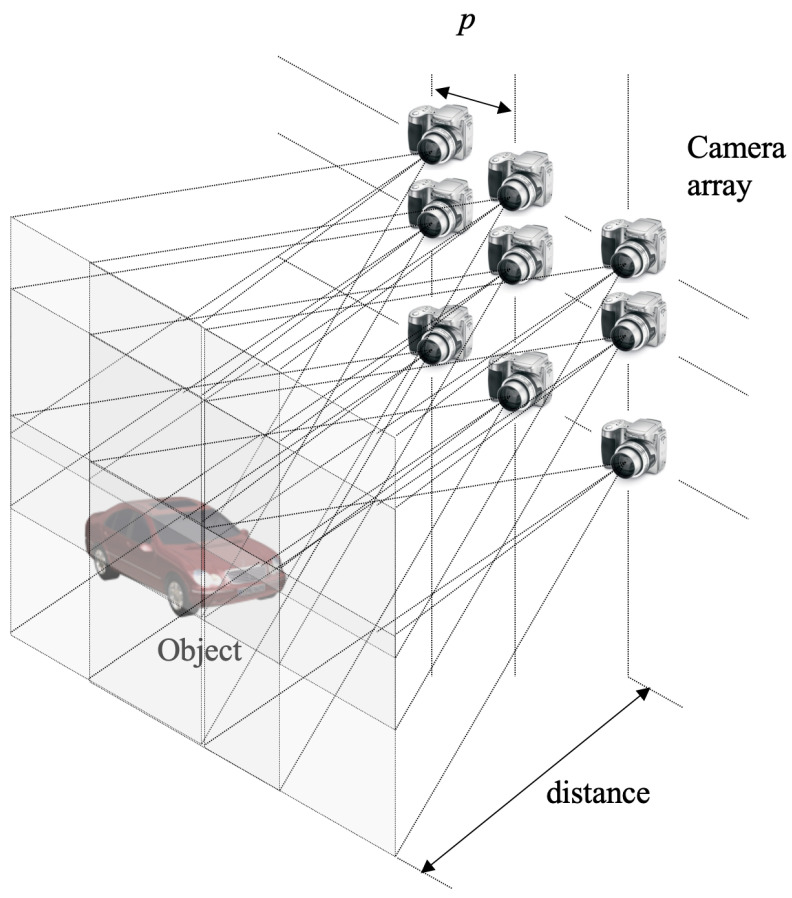
Synthetic aperture integral imaging (SAII).

Figure 11 shows the concept of synthetic aperture integral imaging. In the camera array, each camera is positioned at a fixed location with a uniform pitch *p* between cameras. Since each elemental image has the same resolution as the camera, high-resolution elemental images are obtained. By applying VCR to these high-resolution elemental images, a 3D image can be generated.

**Figure 12:**
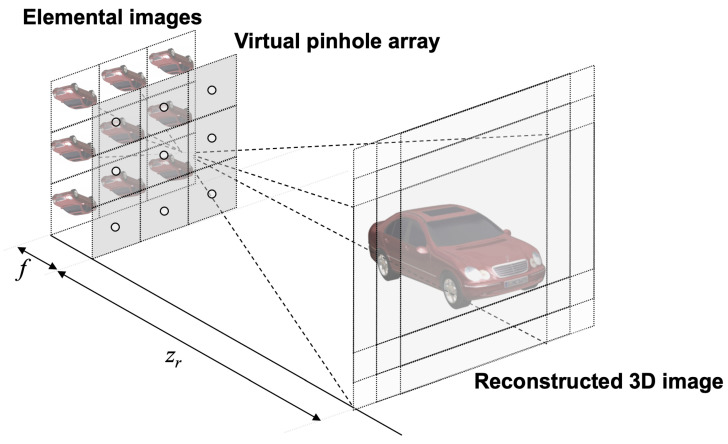
Volumetric computational reconstruction (VCR) of integral imaging.

Figure 12 explains the VCR process in integral imaging. All elemental images are back-projected onto the reconstruction plane through a virtual pinhole array. The distance between the elemental images and the virtual pinhole array corresponds to the focal length *f* of the camera in SAII, while the reconstruction depth $z_{r}$ is the distance between the reconstruction plane and the virtual pinhole array. In the VCR process, the shifting pixel values are applied to overlap all elemental images on the reconstruction plane, as given by [27,28].

(7) $$S_{i}=\left\lceil \frac{N_{x}fp}{c_{x}z_{r}}\times \left(i-1\right)\right\rfloor ,\;\mathrm{for}\;i=1,2,\ldots ,L_{x}$$

(8) $$S_{j}=\left\lceil \frac{N_{y}fp}{c_{y}z_{r}}\times \left(j-1\right)\right\rfloor ,\;\mathrm{for}\;j=1,2,\ldots ,L_{y}$$

(9) $$I\left(x,y,z_{r}\right)=\frac{1}{O(x,y,z_{r})}\sum_{i=1}^{L_{x}}\sum_{j=1}^{L_{y}}I_{ij}\left(x+S_{i},y+S_{j}\right)$$

where i,j are the indices of the elemental images, $L_{x},L_{y}$ are the total number of elemental images in the *x* and *y* directions, $N_{x},N_{y}$ are the number of pixels for each elemental image, *f* is the focal length of the camera, *p* is the pitch between the cameras, $c_{x},c_{y}$ are the image sensor size, $z_{r}$ is the reconstruction depth, ⌈·⌋ denotes the rounding operator, $I_{ij}$ is the $i^{th}$ column and $j^{th}$ row elemental image, and $O(x,y,z_{r})$ is the overlapping matrix at the reconstruction depth $z_{r}$.

Applying Equations (7)–(9) to our proposed method, the reconstructed 3D image can be expressed as

(10) $$\tilde{I}\left(x,y,z_{r}\right)=\frac{1}{O(x,y,z_{r})}\sum_{i=1}^{L_{x}}\sum_{j=1}^{L_{y}}{\tilde{s}}_{dij}\left(x+S_{i},y+S_{j}\right),$$

where ${\tilde{s}}_{dij}$ denotes the $i^{th}$ column and $j^{th}$ row of the decrypted elemental image.

**Figure 13:**
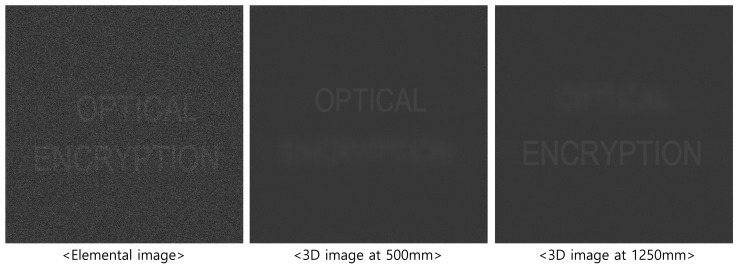
Three-dimensional reconstruction results for the proposed method using 50 diagonal entries in Σ of SVD are used.

**Figure 14:**
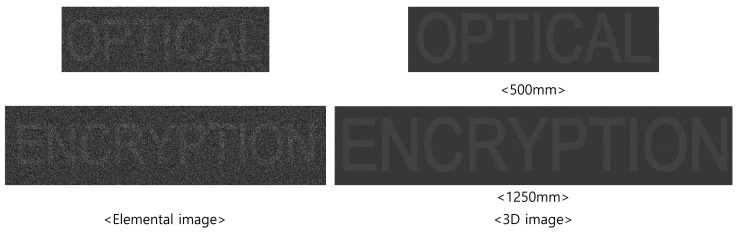
Enlarged images of Figure 13.

Figure 13 and Figure 14 present the 3D reconstruction results and their enlarged views, respectively. In this experiment, 10(H) × 10(V) elemental images were recorded by SAII, each with 1000(H) × 1000(V) pixels. The objects “OPTICAL” and “ENCRYPTION” were positioned at 500 and 1250 mm, respectively. As shown in Figure 13, the elemental image obtained by DRPE and SVD with 50 diagonal entries in Σ exhibits lower visual quality compared to the 3D reconstruction result. The enlarged images in Figure 14 further demonstrate that the 3D images contain less noise than the elemental image.

To demonstrate the feasibility of the proposed method, optical experiments with real objects are conducted in the following section. Additionally, numerical analyses are performed using the peak signal-to-noise ratio (PSNR), structural similarity index measure (SSIM), and peak sidelobe ratio (PSR) of the correlation results.

## 5. Experimental Results

### 5.1. Experimental Setup

**Figure 15:**
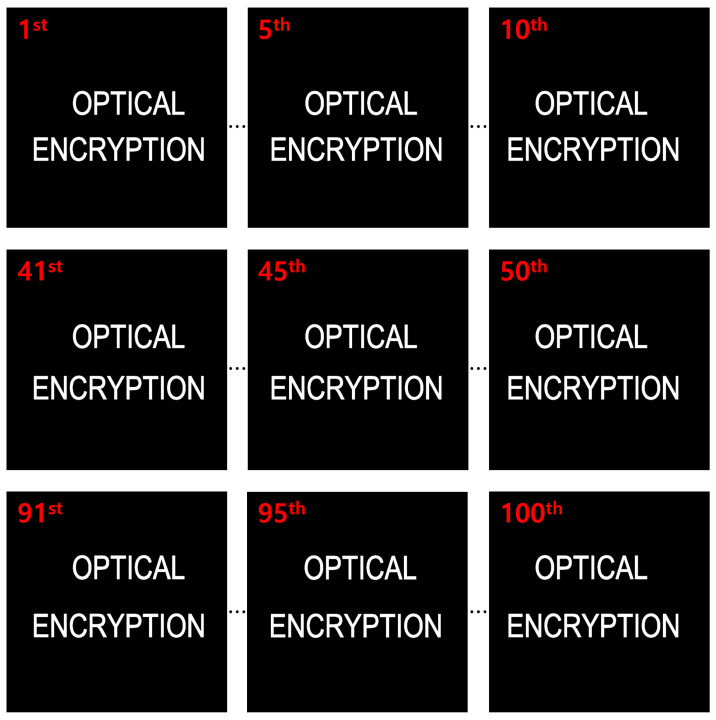
Elemental images used in the simulation.

In the simulation, we used 10(H) × 10(V) elemental images, each with a resolution of 1000(H) × 1000(V) pixels. The focal length of the camera was set to 50 mm, the pitch between cameras was 3.6 mm, and the sensor size was 36(H) × 36(V) mm. Two planar objects, labeled “OPTICAL” and “ENCRYPTION”, were placed at depths of 500 and 1250 mm, respectively. Figure 15 shows the elemental images used in the simulation.

**Figure 16:**
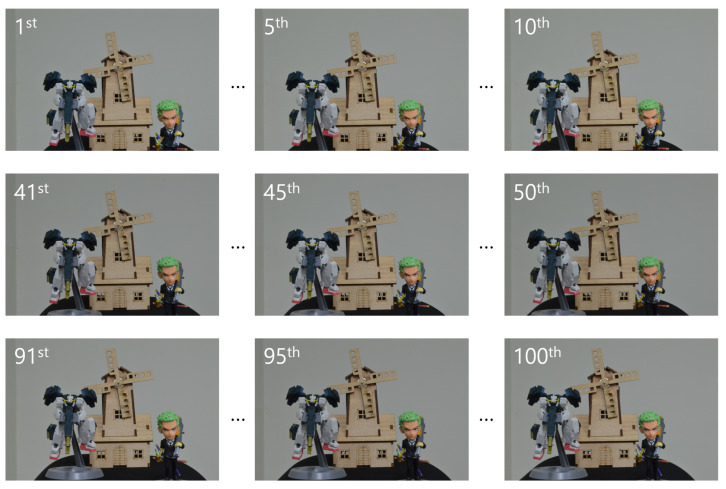
Elemental images used in the optical experiment.

In the optical experiment, we also used 10(H) × 10(V) elemental images, but with a higher resolution of 1504(H) × 1000(V) pixels. These images were recorded using SAII, with a camera focal length of 50 mm, a pitch of 2 mm between cameras, and a sensor size of 36(H) × 24(V) mm. Three different objects were used: object 1 (a figure), object 2 (a robot), and object 3 (a windmill), located at depths of 290, 350, and 400 mm, respectively. Figure 16 presents the elemental images used in the optical experiment.

### 5.2. Results and Discussions

**Figure 17:**
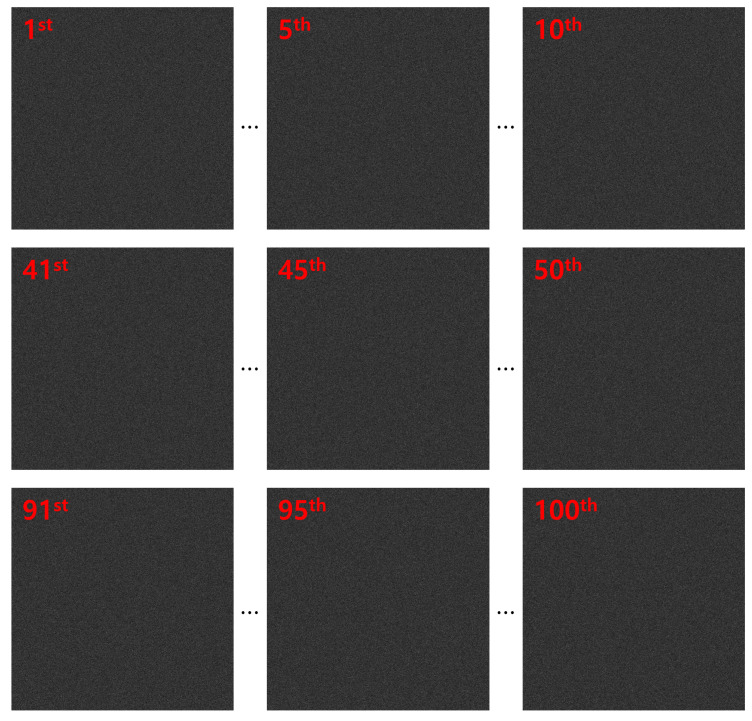
Encrypted elemental images by DRPE in simulation.

**Figure 18:**
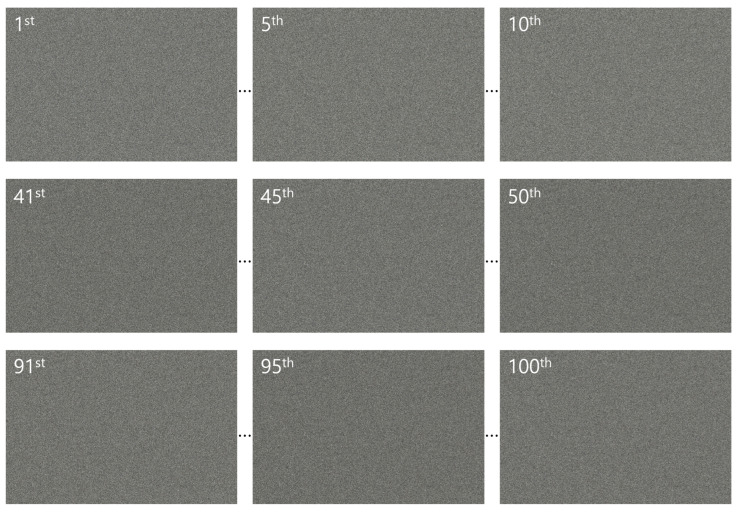
Encrypted elemental images by DRPE in the optical experiment.

**Figure 19:**
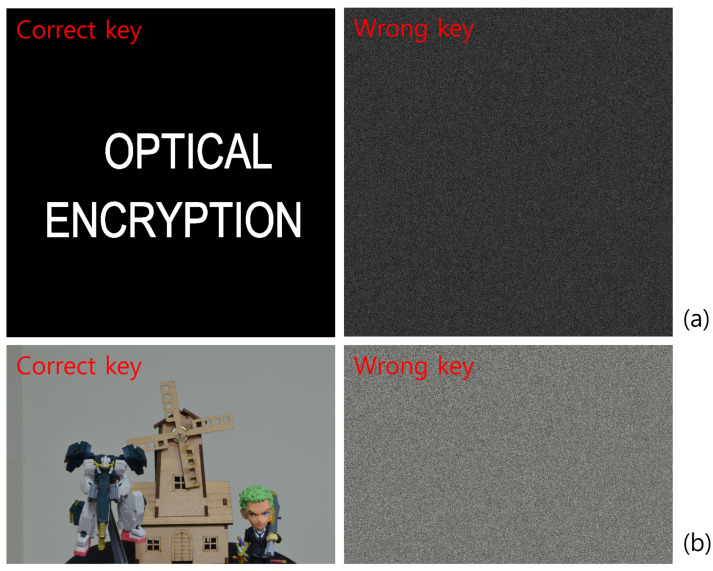
Decrypted images with correct and incorrect key information. (**a**) Simulation and (**b**) optical experiment.

Encrypted elemental images were generated using Equation (1) for both the simulation and optical experiment, as shown in Figure 17 and Figure 18. These encrypted images appear noise-like and are unrecognizable. Using Equation (2) and the correct key information, the decrypted images were obtained, as shown in Figure 19. When the correct key is used, the decrypted images match the original images. In contrast, using incorrect key information results in noise-like decrypted images, thereby demonstrating the security of the DRPE system.

However, the size of the encrypted data is significantly large. For example, in the simulation, the total size of the encrypted data is calculated as 1000 × 1000 × 3 × 2 × 100 = 600,000,000 bytes, where 3 represents the RGB channels, 2 accounts for the complex values, and 100 is the number of elemental images. This large data size poses challenges for wireless transmission. To address this issue, we apply SVD-based compression to DRPE using Equations (3)–(5).

**Figure 20:**
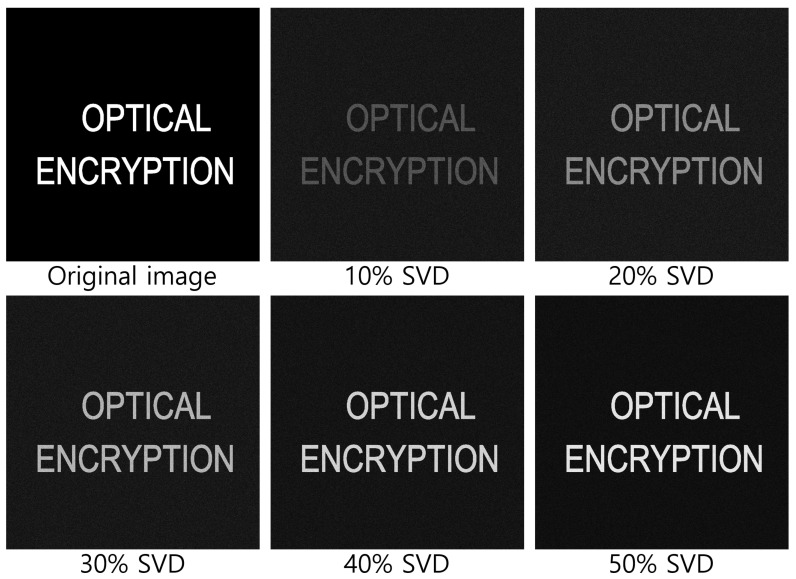
Decrypted images for simulation using SVD with various diagonal entries in Σ (e.g., 10% = 1000 × 0.1 = 100).

**Figure 21:**
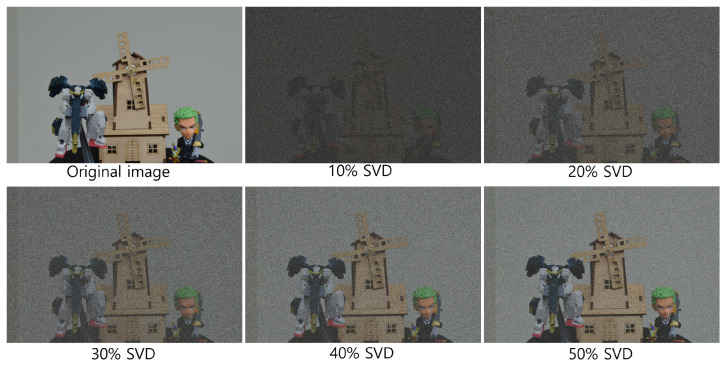
Decrypted images for the optical experiment using SVD with various diagonal entries in Σ (e.g., 10% = 1000 × 0.1 = 100).

Figure 20 and Figure 21 show the decrypted images obtained using SVD with various numbers of diagonal entries in Σ. The percentage indicates the ratio of used diagonal entries to the total rank. For instance, in the simulation, a 10% SVD corresponds to 1000 × 0.1 = 100 diagonal entries. As the number of diagonal entries increases, the visual quality of the decrypted images improves.

To evaluate the performance quantitatively, we compute the peak signal-to-noise ratio (PSNR) and structural similarity index measure (SSIM), as shown in Table 1 and Table 2. The PSNR values increase linearly with the number of diagonal entries for both simulation and optical experiments. Interestingly, the SSIM values for 20 diagonal entries are higher than those for 50–100 entries in some cases. This is due to background noise suppression, which is more effective with fewer singular values, as seen in Figure 20 and Figure 21.

**Figure 22:**
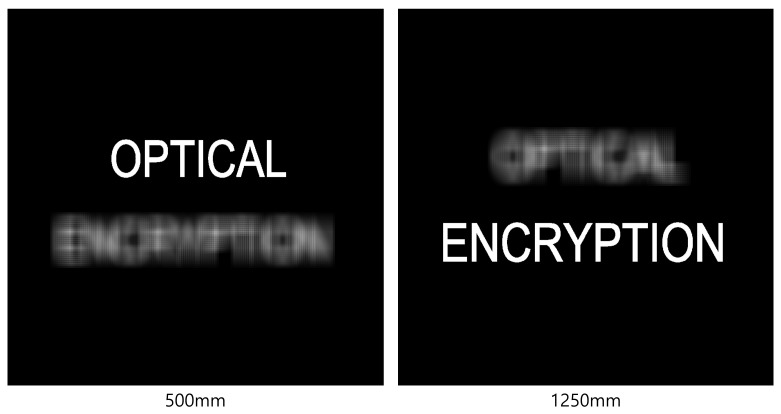
Three-dimensional reconstruction results from simulation.

**Figure 23:**
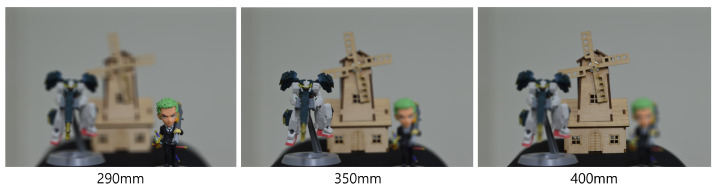
Three-dimensional reconstruction results from the optical experiment.

To further enhance security, we incorporate integral imaging and VCR into the DRPE system. Figure 22 and Figure 23 show the 3D reconstruction results for the simulation and the optical experiment, respectively, using Equations (7)–(9). When both the key information and the correct reconstruction depth are known, the original information can be successfully retrieved.

**Figure 24:**
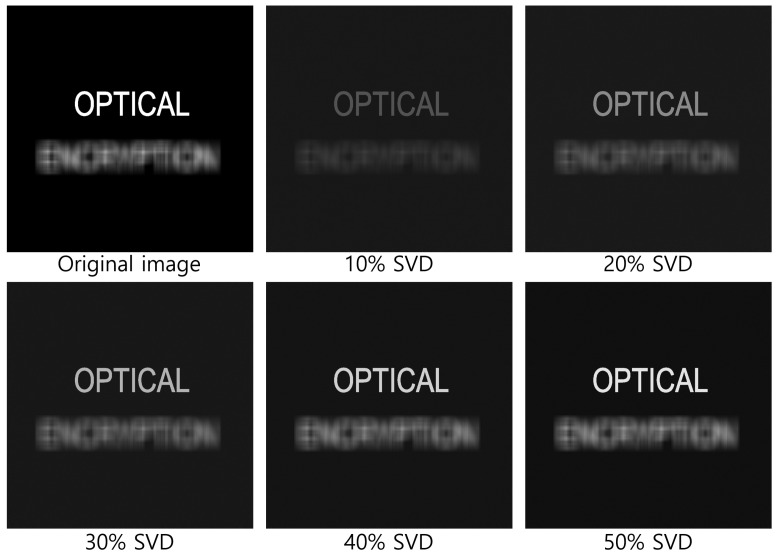
Three-dimensional reconstruction results at 500 mm from simulation with various percentages of SVD.

**Figure 25:**
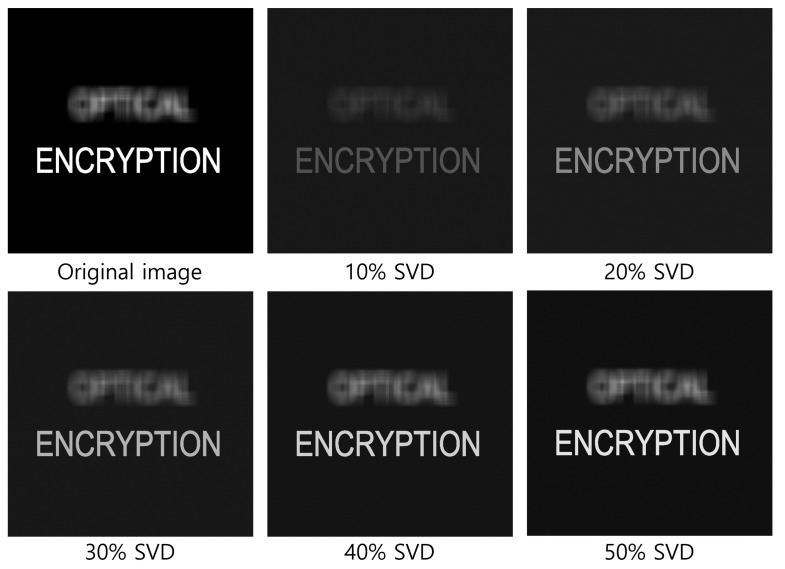
Three-dimensional reconstruction results at 1250 mm from simulation with various percentages of SVD.

**Figure 26:**
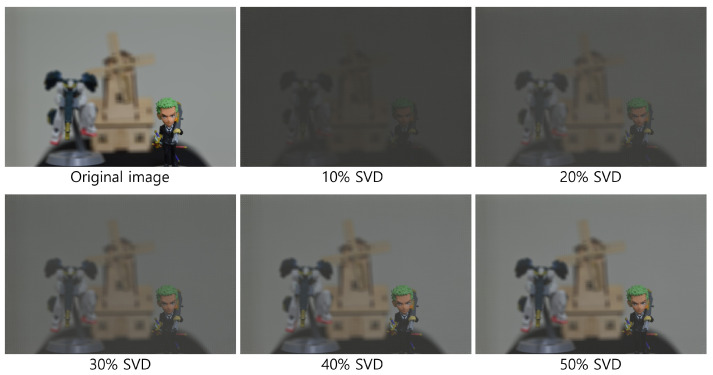
Three-dimensional reconstruction results at 290 mm from the optical experiment with various percentages of SVD.

**Figure 27:**
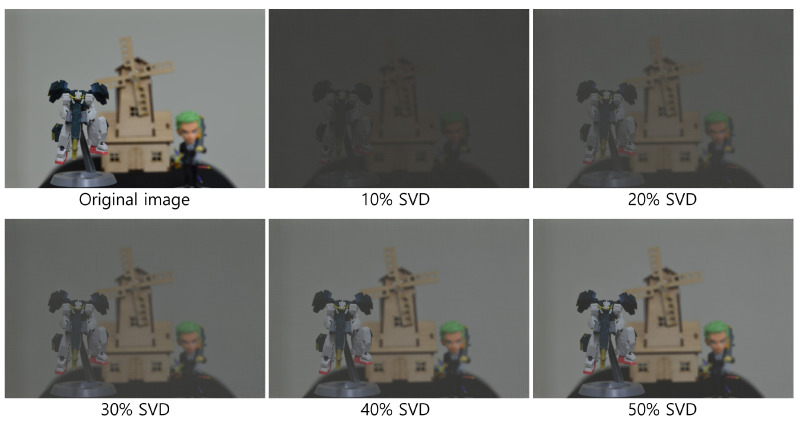
Three-dimensional reconstruction results at 350 mm from the optical experiment with various percentages of SVD.

**Figure 28:**
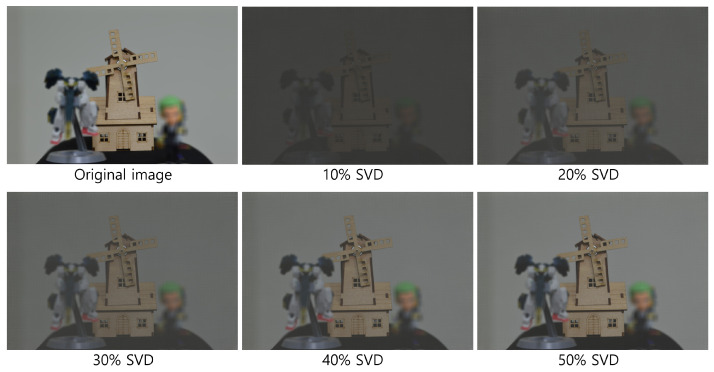
Three-dimensional reconstruction results at 400 mm from the optical experiment with various percentages of SVD.

Moreover, by applying SVD to 3D DRPE using Equation (10), we can reconstruct the correct information with significantly reduced data, as shown in Figure 24, Figure 25, Figure 26, Figure 27 and Figure 28.

As shown in Figure 24 and Figure 25, even when a small number of diagonal entries is used in the SVD process, the objects labeled “OPTICAL” and “ENCRYPTION” can still be clearly reconstructed at depths of 500 and 1250 mm, respectively. Furthermore, as the percentage of diagonal entries increases, the brightness and visual quality of the reconstructed images improve.

Figure 26, Figure 27 and Figure 28 show the 3D reconstruction results for the optical experiment. Object 1 (figure), object 2 (robot), and object 3 (windmill) are successfully focused at depths of 290, 350, 400 mm, respectively, using VCR with various numbers of diagonal entries. Notably, even with a low number of diagonal entries, the objects can still be reconstructed with acceptable quality. These results confirm the effectiveness of the proposed compression method.

To quantitatively compare the 2D and 3D results, PSNR and SSIM values for various compression ratios, as defined in Equation (6), are presented in Table 3 and Table 4. In the simulation with a compression ratio of 4.9975, the PSNR of the 3D image at 500 mm is 3 dB higher than that of the 2D image. Similarly, in the optical experiment, for compression ratios of 1.6658, 1.2494, and 0.9995, the PSNR values for the 3D images at 290, 350, and 400 mm are more than 3 dB higher than those of the corresponding 2D images.

These results clearly demonstrate that the proposed method significantly enhances visual quality, compression efficiency, and security. Furthermore, in the simulation, all SSIM values for the 3D images at 500 and 1250 mm are at least twice as high as those for the 2D images. In the optical experiment, the SSIM values for 3D images at 290, 350, and 400 mm are at least six times higher than those for the 2D images. These findings confirm the feasibility and effectiveness of the proposed method.

To further demonstrate that 3D information can serve as an additional key in the proposed method, the $k^{th}$ law nonlinear correlation filter [34] is applied, and the peak sidelobe ratio (PSR) [34] is calculated for various reconstruction depths and different numbers of diagonal entries in the SVD. The $k^{th}$ law nonlinear correlation filter is defined as [34]

(11) $$c\left(x\right)=F^{-1}\left[{\left\{\left|I_{ref}\left(\mu \right)\right|\left|{\tilde{I}}_{z_{r}}\left(\mu \right)\right|\right\}}^{k}e^{\left\{\phi_{ref}\left(\mu \right)-{\tilde{\phi }}_{z_{r}}\left(\mu \right)\right\}}\right],$$

where $I_{ref}\left(\mu \right)$ is the Fourier transform of the reference 3D image, as shown in Figure 22 and Figure 23, $\phi_{ref}\left(\mu \right)$ is its phase, ${\tilde{I}}_{z_{r}}\left(\mu \right)$ is the Fourier transform of the reconstructed 3D image as shown in Figure 24, Figure 25, Figure 26, Figure 27 and Figure 28, and ${\tilde{\phi }}_{z_{r}}\left(\mu \right)$ is its phase. The parameter *k* is a nonlinear factor, and in this paper, we use k=0.3.

The PSR is a metric that quantifies the strength of the correlation peak and is defined as [34]

(12) $$PSR=\frac{\max\left[c(x)\right]-\bar{c}\left(x\right)}{\sqrt{\mathrm{var}\left[c(x)\right]}},$$

where $\bar{c}\left(x\right)$ is the mean of the correlation output c(x).

**Figure 29:**
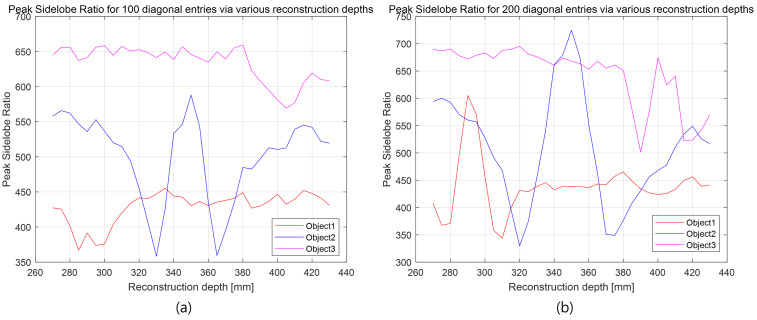
Peak sidelobe ratio (PSR) at various reconstruction depths: (**a**) 100 diagonal entries and (**b**) 200 diagonal entries.

Figure 29 shows the PSR values for reconstruction depths using 100 and 200 diagonal entries. As shown in Figure 29a, object 1 cannot be detected at its correct depth (i.e., 290 mm) when only 100 diagonal entries are used. Similarly, in Figure 29b, object 3 is not detected at its correct depth (i.e., 400 mm) with 200 diagonal entries.

**Figure 30:**
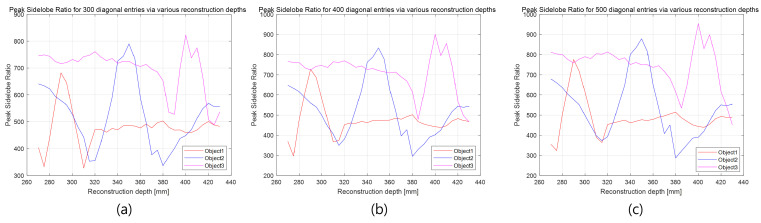
Peak sidelobe ratio (PSR) at various reconstruction depths: (**a**) 300 diagonal entries, (**b**) 400 diagonal entries, and (**c**) 500 diagonal entries.

In contrast, all objects are successfully detected at their respective depths in Figure 30, where 300, 400, and 500 diagonal entries are used. This confirms that a sufficient number of singular values is necessary for accurate 3D object reconstruction and correlation. In this study, the original elemental images consist of 10(H) × 10(V) images with RGB channels and a resolution of 1504(H) × 1000 pixels. The total data size is 1504 × 1000 × 3 × 100 = 451,200,000 bytes. Using our proposed method with 300 diagonal entries, the compressed data size becomes 300 × (1504 + 1000 + 1) × 3 × 100 = 225,450,000 bytes. Thus, the compression ratio, as defined in Equation (6), is approximately 2. These results demonstrate that our proposed method can simultaneously improve visual quality, compression ratio, and security.

## 6. Conclusions

In this paper, we proposed a novel compression method for double random phase encryption (DRPE) by incorporating singular value decomposition (SVD) and volumetric computational reconstruction (VCR). Since the encrypted data generated by DRPE are complex-valued, the data size becomes twice that of the original primary data. Moreover, if the key information is exposed, the primary data can be easily recovered, posing a security risk. Additionally, when a small number of diagonal entries is used in SVD, the visual quality of the decrypted data deteriorates due to information loss. To address these issues, we employed VCR based on integral imaging, which enables the use of 3D information as an additional encryption key. The proposed method not only enhances data security but also improves visual quality and compression efficiency simultaneously. Experimental results, including optical experiments, demonstrate that our method significantly reduces the data size while maintaining acceptable visual quality. However, several challenges remain. When applying SVD for compression, determining the optimal number of diagonal entries is crucial. Using too few diagonal entries results in reduced brightness and degraded image quality. These limitations may be addressed in future work by exploring optimization strategies and integrating photon-counting integral imaging into the proposed framework. In summary, the proposed method effectively improves the visual quality, compression ratio, and security of DRPE. Future research will focus on optimizing the number of singular values and enhancing performance through advanced imaging techniques.

## Figures and Tables

**Table 1:** Peak signal-to-noise ratio (PSNR) for compression of 2D images.

Diagonal Entries	50	60	70	80	90
**Simulation**	13.8468	14.0089	14.1714	14.3333	14.4963
**Optical experiment**	8.7043	8.9966	9.2637	9.5104	9.7394
**Diagonal entries**	**100**	**200**	**300**	**400**	**500**
**Simulation**	14.6598	16.3579	18.2157	20.3167	22.7630
**Optical experiment**	9.9537	11.6613	13.0951	14.6158	16.3871

**Table 2:** Structural similarity index measure (SSIM) for compression of 2D images.

Diagonal Entries	50	60	70	80	90
**Simulation**	0.0148	0.0156	0.0168	0.0182	0.0198
**Optical experiment**	0.0639	0.0613	0.0596	0.0585	0.0578
**Diagonal entries**	**100**	**200**	**300**	**400**	**500**
**Simulation**	0.0215	0.0376	0.0490	0.0585	0.0705
**Optical experiment**	0.0573	0.0631	0.0786	0.1019	0.1356

**Table 3:** Peak signal-to-noise ratio (PSNR) comparison for compression of 2D and 3D images.

Compression Ratio	4.9975	2.4988	1.6658	1.2494	0.9995
**Simulation (2D)**	14.6598	16.3579	18.2157	20.3167	22.7630
**Simulation (500 mm)**	**17.6660**	18.8990	20.3792	22.1842	24.4060
**Simulation (1250 mm)**	16.4376	18.0563	19.8175	21.8214	24.1811
**Optical experiment (2D)**	9.9537	11.6613	13.0951	14.6158	16.3871
**Optical experiment (290 mm)**	11.3219	13.9279	**16.6633**	**19.6083**	**22.9250**
**Optical experiment (350 mm)**	11.2243	13.8011	**16.5139**	**19.4433**	**22.7514**
**Optical experiment (400 mm)**	11.2108	13.7836	**16.4958**	**19.4259**	**22.7368**

**Table 4:** Structural similarity index measure (SSIM) comparison for compression of 2D and 3D images.

Compression Ratio	4.9975	2.4988	1.6658	1.2494	0.9995
**Simulation (2D)**	0.0215	0.0376	0.0490	0.0585	0.0705
**Simulation (500 mm)**	0.0890	0.1127	0.1267	0.1372	0.1495
**Simulation (1250 mm)**	0.0775	0.1010	0.1152	0.1258	0.1382
**Optical experiment (2D)**	0.0573	0.0631	0.0786	0.1019	0.1356
**Optical experiment (290 mm)**	0.5240	0.6058	0.6728	0.7350	0.7947
**Optical experiment (350 mm)**	0.5176	0.6053	0.6760	0.7405	0.8020
**Optical experiment (400 mm)**	0.5216	0.6094	0.6809	0.7455	0.8062

## Data Availability

Data are contained within the article.

## Abbreviations

The following abbreviations are used in this manuscript:

DRPE	Double random phase encryption
PSNR	Peak signal-to-noise ratio
PSR	Peak sidelobe ratio
SSIM	Structural similarity index measure
SVD	Singular value decomposition
VCR	Volumetric computational reconstruction
